# Determinants of exercise peak arterial blood pressure, circulatory power, and exercise cardiac power in a population based sample of Finnish male and female aged 30 to 47 years: the Cardiovascular Risk in Young Finns Study

**DOI:** 10.1186/1471-2261-14-35

**Published:** 2014-03-13

**Authors:** Janne Hulkkonen, Heikki Aatola, Kristiina Pälve, Terho Lehtimäki, Nina Hutri-Kähönen, Jorma SA Viikari, Olli T Raitakari, Mika Kähönen

**Affiliations:** 1 Department of Clinical Physiology, Tampere University Hospital, Tampere, Finland; 2 Department of Clinical Chemistry, Fimlab laboratories, Tampere University Hospital, Tampere, Finland; 3 Research Centre of Applied and Preventive Cardiovascular Medicine, University of Turku, Turku, Finland; 4 The School of Medicine, University of Tampere, Tampere, Finland; 5 Department of Paediatrics, Tampere University Hospital, Tampere, Finland; 6 Department of Medicine, University of Turku, Turku, Finland; 7 Department of Clinical Physiology and Nuclear Medicine, Turku University Hospital, Turku, Finland; 8 Fimlab laboratories, P.O. Box 66, Tampere, FI 33101, Finland

**Keywords:** Aerobic capacity, Peak oxygen consumption, Population, Cardiopulmonary exercise test, Oxygen pulse, Circulatory power, Exercise cardiac power, Blood pressure

## Abstract

**Background:**

Novel parameters derived from peak maximal oxygen uptake (VO2) and exercise arterial blood pressure, such as peak circulatory power (CP) and exercise cardiac power (ECP), can be used in the risk assessment of cardiovascular disease and stroke. However, the determinants of these factors are poorly characterized in the general population.

**Methods:**

We assessed peak arterial blood pressure, CP and ECP with standardized cardiopulmonary exercise test (CPET) on 281 female and 257 male participants of the Cardiovascular Risk in Young Finns Study. The subjects were aged 30–47 years. Peak VO2 as well as systolic and diastolic arterial blood pressures were measured to calculate peak mean arterial pressure, CP and ECP. These parameters were assessed for correlation with sex, age, height, weight, waist-to-hip ratio, smoking, physical activity index (PAI), fasting insulin and glucose levels as well as the use of antihypertensive treatment.

**Results:**

Sex, age and weight explained 36% of the variation in peak systolic blood pressure, and these factors in combination with height and the use of antihypertensive treatment explained 13% of the variation in peak diastolic blood pressure. Sex, height, weight, waist-to-hip ratio, PAI and smoking explained 49% − 52% of the variation in peak CP. Sex, age, height, weight, waist-to-hip ratio, PAI, smoking and insulin levels explained 21% − 49% of variation in ECP.

**Conclusions:**

Subject demographics and lifestyle-related factors should be taken into account when exercise blood pressure response, CP and ECP are used to evaluate patients’ cardiac function in CPET.

## Background

Peak oxygen uptake (VO2) is a measure of the of the cardiovascular system’s functional capacity [1]. Peak VO2, exercise blood pressure (BP) response and several other parameters related to cardiovascular fitness can be assessed easily with a cardiopulmonary exercise test (CPET) [1-6]. Low maximal aerobic capacity predicts, to an extent, cardiovascular events, morbidity and mortality in later life [7-10]. In addition to peak systolic blood pressure (SBP), peak diastolic blood pressure (DBP), mean arterial blood pressure (MAP), and oxygen pulse (VO2/heart rate [HR]), some novel parameters such as peak circulatory power (CP) and exercise cardiac power (ECP) have been suggested to improve the prognostic accuracy of a CPET [5,11-16].

CP, expressed as peak VO2 multiplied by peak SBP or MAP, represents the volume of O2 added to the mixed venous blood by the lungs and transferred to systemic arterial circulation, against a pressure gradient, by the heart [5,17]. As the gradient through which the O2 is transferred by the heart is SBP as opposed to MAP, CP also relates to an instantaneous peak work of the cardiac cycle [5,17]. It has also been suggested that if CP is calculated as the product of peak VO2 and MAP rather than SBP, it may rule in the cardiac afterload more accurately [11,17].

Another non-invasive surrogate for cardiac power output is exercise cardiac power (ECP), which is the product of peak VO2 (expressed as a percent of predicted) and peak SBP. ECP combines exercise SBP as an estimate of afterload with peak VO2 as an estimate of peak cardiac output neglecting preload [6,13]. When ECP is normalized against subject demographics by expressing peak VO2 as a percentage of predicted VO2 (i.e. by using reference value equations), the prognostic value of each indicator is improved and ECP serves as an independent predictor of mortality and morbidity in congestive heart failure [6]. The integration of peak VO2 with SBP by analyzing the ratio of these parameters improves the predictive power of stroke-risk assessment as well [13].

As mentioned above, peak VO2, peak BP response, and several parameters derived from these factors have a marked prognostic significance. However, the determinants and levels of CP and ECP have not been characterized previously in a general population. Therefore, we carried out CPETs for a sample of 538 subjects drawn from the Cardiovascular Risk in Young Finns Study and explored peak SBP, DBP, MAP, CP and ECP in relation to sex, age, height, weight, waist-to-hip ratio, smoking, physical activity index (PAI), fasting insulin and glucose, and the use of antihypertensive treatment.

## Methods

### Subjects

The Cardiovascular Risk in Young Finns Study is an on-going follow-up study on the precursors of atherosclerosis in Finnish children and adolescents. The first cross-sectional survey was conducted in 1980 when 3,596 participants aged 3, 6, 9, 12, 15 and 18 years were randomly chosen from the national population register to represent each (geographical) area in Finland [18]. The sample was fairly representative of the entire Finnish population. The fourth follow-up study was conducted in 2007 with 2204 participants. Of those, 538 individuals (257 males and 281 females) aged from 30 to 47 years volunteered for a cardiopulmonary exercise test (CPET) during the years 2007 to 2009. The characteristics of the participants in the 2007–2009 cross-section are presented in Table 1. The study was approved by the ethics committee of the Tampere University Hospital, and all subjects gave their written informed consent.

**Table 1:** Subject characteristics

**Parameter**	**Female**	**Male**
	**(n = 281)**	**(n = 257)**
Age (y)	38.9 ± 5.0	38.2 ± 5.1
Height (cm)	166 ± 5.4	180 ± 6.4
Weight (kg)	70.5 ± 13.6	86.9 ± 14.6
BMI (kg/m^2^)	25.7 ± 5.1	26.9 ± 4.2
Waist-to-hip ratio	0.85 ± 0.06	0.94 ± 0.076
SBP (mmHg)	123 ± 14	133 ± 14
DBP (mmHg)	79 ± 9	84 ± 11
MAP (mmHg)	99 ± 10	101 ± 10
Fasting glucose (mmol/L)	5.2 ± 0.69	5.5 ± 0.68
Fasting Insulin (mmol/L)	8.6 ± 7.0	9.3 ± 10.9
PAI	8.8 ± 1.8	8.7 ± 2.0
FEV1/FVC (%)	80.7 ± 5.5	78.8 ± 5.9
Antihypertensive treatment (%)	5.0	4.3
Smoking (%)	11.0	15.2

Values are mean ± SD if not otherwise indicated.

BMI: body mass index; SBP: systolic blood pressure; DBP: diastolic blood pressure; MAP: mean arterial pressure; PAI: physical activity index; FEV1/FVC: ratio of forced expiratory capacity in one second and forced vital capacity.

### Clinical characteristics and risk factors

Height, weight as well as waist and hip circumferences were measured, and the waist-to-hip ratios were calculated. Smoking habits were assessed with a questionnaire, and smoking was modeled as a dichotomous variable (smoking/non-smoking) defined as regular cigarette smoking on a daily basis or more frequently. To measure physical activity, a short self-report questionnaire was administered for the subjects. The questions involved the intensity of physical activity, frequency of vigorous physical activity, hours spent on vigorous physical activity, average duration of a physical activity session and participation in organized sports. A physical activity index (PAI) ranging from 5 to 15 was calculated as a sum score of these items. The lowest scores indicate passive, and the highest scores indicate active. The original description of the method has been published previously [19]. Plasma glucose and insulin concentrations were measured using standard methods [20]. The demographic, clinical and laboratory data on the subjects are presented in Table 1.

### Cycle ergometry, spirometry and gas measurements

Exercise tests were performed on electronically braked cycle ergometers (Lode Corival 906900, Lode BV, Groningen, Netherlands) according to the American Thoracic Society (ATS) guidelines and the American College of Chest Physicians (ACCP) Joint Statement on Cardiopulmonary Exercise Testing [1]. In brief, after a 10-minute rest and a warm-up period of 10–60 seconds, the subjects performed an incremental test with 1-minute intervals and increments of 15 W/minute for women and 20 W/minute for men until exhaustion limited maximal power output. Otherwise, objective test termination criteria (established in ref. [1]) were applied by the observers. Standard 12-lead electrocardiography (ECG) was recorded during the test (Corina ECG amplifier and CardioSoft acquisition software ver. 4.2, GE Medical Systems, Freiburg, Germany). BP was measured with the cuff method by means of auscultation. The mean arterial pressure was calculated using the formula: (SBP + 2* [DBP])/3 as suggested elsewhere [11]. Peak HR was defined as the maximal heart rate achieved during the exercise. The rating of perceived exertion was obtained using the Borg category scale [21]. In order to assess forced vital capacity (FVC) and forced expiratory volume in the first second (FEV1), a standard flow-volume spirometry measurement was performed with a calibrated spirometer (V-max 29C, SensorMedics, Yorba Linda, CA, USA) according to the ATS and the European Respiratory Society (ERS) Task Force guidelines [22]. Breath-by-breath measurements of VO2 and carbon dioxide output (VCO2) as well as a measurement of ventilatory parameters were performed with computerized analyzers (V-max 29C, SensorMedics, Yorba Linda, CA, USA and Jaeger Oxycon Pro, VIASYS Healthcare GmbH, Hoechberg, Germany). As two different analyzers were used, the linearity of the gas measurement devices was checked prior to the analyses with a metabolic simulator (VacuMed Syringe model 17050 calibration kit, Ventura, CA, USA) at the ventilation levels of 34–90 liters/min. This calibration method has been proven valid [23]. The correlation between expected and measured values at this test range suggested good linearity for both devices (R^2^ values for VO2 and VCO2 measurements > 0.99). The inter-instrument difference varied from 1% to 3% for tidal volume measurements, VO2 and VCO2 which is within the expected range [1]. After calibration measurements, O2 and CO2 test gases were used daily in order to check the reproducibility of the measurements. The final tabular and graphic averaged data were collected at 30-second intervals as recommended [1]. Peak VO2 was determined as the highest VO2 during the last 30-second averaged interval. Peak work rate (WR) was calculated by adding the WR during the last full one-minute interval to the fraction of the work performed over the final, exhaustion-limited (interrupted) interval. The expected peak HR was calculated using the formula 220 minus age (years). Standard published equations were applied to generate predicted values for peak VO2 [24-26]. CP was calculated as the product of peak VO2 (expressed in mL/kg/min) and peak SBP (expressed as mmHg) at the last BP measurement [5,12,14], or, alternatively, as the product of peak VO2 (mL/kg/min) and MAP (mmHg) [11,16]. ECP [6] was originally calculated as the product of peak SBP (mmHg) and peak VO2 expressed as a percentage of the predicted peak VO2 (%) by the equation provided by Wassermann et al. [26]. This predictive equation for VO2 rules in age, weight and sex. In order to improve the generalization of the results, we also calculated the percentage of predicted VO2 using alternative reference value equations, namely those from the European SHIP- study [25], which take into account age, sex, and body mass index (BMI), and the North American equation by Jones et al. [24], which stratifies the predicted VO2 by age, sex and height. As there is one previous key study that integrates SBP with peak VO2 by the ratio of these parameters, we also explored these figures [13].

While all subjects carried out the cycle ergometer test and reached the objective maximum (based on respiratory quotient > 1.0, peak HR at the range of expected maximum of HR ± 10 or Borg category scale >19), we decided to exclude gas measurement data for 8 subjects (1.5% of tested individuals). The reason for this was technical error such as mask leakage or computer failure. For one subject, the gas measurements were interrupted, as the subject could not tolerate the mask.

### Statistical analysis

Numerical values are presented as means ± standard deviation (SD). Statistical analyses were initially carried out using Statistica for Windows 6.0 (StatSoft Inc., Tulsa, Oklahoma, USA), and PASW Statistics 18.0 for Windows (SPSS Inc., Chicago, Illinois, USA), with Kruskall-Wallis analysis of variance and the Mann–Whitney *U*-test. For correlation analysis, the Pearson product–moment correlation coefficient was calculated. After non-parametric analyses variables potentially explaining peak SBP, DBP, CP, and ECP were entered into a multivariable linear regression model. The original covariates in the models were sex, age, height, weight, waist-to-hip ratio, PAI, smoking, fasting insulin and glucose, and the anamnestic use of antihypertensive treatment. Values for insulin and glucose were log-transformed before analyses, because of skewed distributions. P-values less than 0.05 were considered significant in all analyses.

## Results

### Determinants of peak aerobic capacity, SBP, DBP, MAP, CP and ECP

The peak (mean ± SD) VO2 values for the whole cohort were 2397 ± 799 mL/min (30.6 ± 8.49 mL/kg/min). Mean WR was 209 ± 62.5 W. Mean peak SBP was 201 ± 27 mmHg, mean DBP 92 ± 13 mmHg, and mean MAP 129 ± 15 mmHg. Mean peak CP of the cohort was 6195 ± 1999 mL/min/kg*mmHg when expressed as the product of VO2 and SBP and 3914 ± 1147 mL/min/kg*mmHg when expressed as the product of VO2 and MAP. Mean ECP was 19801 ± 5047 %mmHg when the VO2 percentage of the predicted value was calculated using the reference value equation provided by Wassermann et al. [26], 20069 ± 5083 %mmHg when the equation from the SHIP-study [25] was applied, and 19594 ± 5234 %mmHg when the equation by Jones et al. [24] was applied. VO2/SBP [13] was 11.9 ± 3.67 mL/mmHg. These figures as well as those representing aerobic capacity (VO2 expressed as % of the predicted value by Wasserman et al. [26] and Koch et al. [25]) are presented separately for female and male subjects in Table 2. Determinants of Peak VO2, SBP, DBP, MAP, CP, and ECP are given in Table 3. In these tables, CP is presented as the product of SBP and peak VO2, or alternatively, as the product of MAP and peak VO2. ECP is expressed as the product of the percentage of the predicted VO2 value with three different reference value equations [24-26], all of which distinctly stratify subject demographics (see Methods). Moreover, the ratio of peak VO2 and SBP is presented [13]. The suggested lower limits (i.e. -2 SD levels) of peak SBP, DBP, MAP, CP, ECP, and VO2/SBP are presented in Table 4.

**Table 2:** Cardiopulmonary exercise test values in men and women

**Parameter**	**Female (n = 281)**	**Male (n = 257)**
	**Mean ± SD**	**Mean ± SD**
VO_2_ (mL/min)	1840 ± 458	3000 ± 632
VO_2_/kg (mL/kg/min)	26.5 ± 6.9	35.0 ± 7.8
SBP (mmHg)	189 ± 23	215 ± 24
DBP (mmHg)	91 ± 13	93 ± 13
MAP (mmHg)	124 ± 14	134 ± 15
CP_SBP_ (mL/min/kg×mmHg)	4990 ± 1330	7490 ± 1780
CP_MAP_ (mL/min/kg×mmHg)	3260 ± 855	4630 ± 988
ECP_Wasserman_ (%mmHg)	19100 ± 5080	20500 ± 4920
ECP_Koch_ (%mmHg)	18800 ± 4900	21400 ± 4930
ECP_Jones_ (%mmHg)	18750 ± 5470	20500 ± 4820
VO_2_/SBP (mL/min/mmHg)	9.8 ± 2.6	14.1 ± 3.3
VO_2_ (% predicted)_Wasserman_	101 ± 24.6	96.0 ± 21.1
VO_2_ (% predicted)_Koch_	99.6 ± 24.0	100 ± 20.9
WR (W)	163 ± 28.9	261 ± 47.4
WR (% predicted)	119 ± 22.7	113 ± 18.9

VO2: oxygen consumption; SBP: systolic blood pressure; DBP: diastolic blood pressure; MAP: mean arterial pressure; CP: circulatory power; ECP: exercise cardiac power; WR: work rate. CP is given as a product of VO2 and systolic blood pressure (CP_SBP_) as well as a product of VO2 and MAP (CP_MAP_). ECP is presented using the alternative VO2 reference value equations provided by Wasserman et al. [26], Koch et al. [25], and Jones et al. [24] and indexed correspondingly in the table as ECP_Wasserman_, ECP_Koch_, and ECP_Jones_.

Values are shown as peak values and as a percentage of the predicted value.

**Table 3:** Determinants of peak VO2, SBP, DBP, MAP, CP, ECP, and VO2/SBP (multivariable regression)

**Parameter**	**VO**_**2**_**,**	**SBP**	**DBP**	**MAP**	**CP**_**SBP**_	**CP**_**MAP**_	**ECP**_**Wasserman**_	**ECP**_**Koch**_	**ECP**_**Jones**_	**VO**_**2**_**/SBP**
	**β ± SE**	**β ± SE**	**β ± SE**	**β ± SE**	**β ± SE**	**β ± SE**	**β ± SE**	**β ± SE**	**β ± SE**	**β ± SE**
Sex	675 ± 77.5‡	18.4 ± 3.6‡	4.3 ± 2.1*	9.1 ± 2.2‡	2300 ± 235‡	1370 ± 140‡	237 ± 764	1770 ± 764*	2080 ± 753†	2.4 ± 0.4‡
Age	−5.5 ± 4.5	0.8 ± 0.2‡	0.5 ± 0.1‡	0.6 ± 0.1‡	10.2 ± 13.6	10.2 ± 8.1	238 ± 44.3‡	153 ± 44.3‡	193 ± 43.7‡	−0.07 ± 0.03†
Height	23.6 ± 3.9‡	−0.12 ± 0.18	−0.26 ± 0.11*	−0.23 ± 0.1*	53.7 ± 11.9‡	24.2 ± 7.2‡	168 ± 38.6‡	117 ± 38.6†	−189 ± 38.0‡	0.12 ± 0.02‡
Weight	10.9 ± 2.2‡	0.42 ± 0.1‡	0.13 ± 0.06*	0.21 ± 0.06†	−29.4 ± 6.6‡	−21.0 ± 4.0‡	−59.7 ± 21.3‡	−46.5 ± 21.3*	136 ± 21.0‡	0.03 ± 0.01*
WHR	32.3 ± 460	28.9 ± 21.4	9.3 ± 12.6	17.2 ± 13.4	−92 ± 1390	531 ± 836	1730 ± 4530	3100 ± 4530	4120 ± 4480	−0.80 ± 2.6
PAI	78.5 ± 11.6‡	0.86 ± 0.54	−0.25 ± 0.32	0.07 ± 0.34	224 ± 35.1‡	118 ± 21.0‡	720 ± 114‡	746 ± 114‡	708 ± 112‡	0.34 ± 0.07‡
Smoking	−228 ± 63.2‡	0.41 ± 2.9	0.52 ± 1.7	0.48 ± 1.8	−619 ± 192†	−389 ± 114‡	−1790 ± 623†	−1870 ± 623‡	−1710 ± 614*	−1.1 ± 0.36†
Fasting insulin	−9.3 ± 3.1‡	0.26 ± 0.15	0.19 ± 0.1	0.25 ± 0.10*	−17.9 ± 9.5	−11.4 ± 6.5	−49.0 ± 30.8	−53.1 ± 30.8	−53.0 ± 30.3	−0.05 ± 0.02†
Fasting glucose	26.8 ± 39.6	−1.1 ± 1.8	−2.0 ± 1.1	−1.9 ± 1.2	158 ± 120	48.9 ± 72.9	381 ± 390	266 ± 390	243 ± 385	0.12 ± 0.23
AHT	−53.6 ± 96.8	2.4 ± 4.5	5.2 ± 2.6*	4.4 ± 2.8	−54.2 ± 294	9.6 ± 175	−327 ± 954	−114 ± 954	−486 ± 940	−0.39 ± 0.56
Whole Model R^2^	67%	36%	13%	26%	52%	49%	21%	21%	28%	49%

VO2: oxygen consumption; SBP: systolic blood pressure; DBP: diastolic blood pressure; MAP: mean arterial pressure; CP circulatory power, ECP: exercise cardiac power; WHR: waist-to-hip ratio; PAI: physical activity index; AHT: antihypertensive treatment; β: regression coefficient for a 1-unit change in parameter; SE: standard error. CP is given as a product of VO2 and SBP (CP_SBP_) as well as a product of VO2 and MAP (CP_MAP_). ECP is presented using the alternative VO2 reference value equations provided by Wasserman et al. [26], Koch et al. [25], and Jones et al. [24] and indexed correspondingly in the table as ECP_Wasserman_, ECP_Koch_, and ECP_Jones_.

*p < 0.05; †p < 0.005, and ‡p < 0.001.

**Table 4:** Lower limits of peak exercise blood pressure, circulatory power and exercise cardiac power

**Parameter**	**Female (n = 281)**	**Male (n = 257)**
	**Mean −2 SD level**	**Mean −2 SD level**
SBP (mmHg)	143	168
DBP (mmHg)	65	67
MAP (mmHg)	95	104
CP_SBP_ (mL/min/kg×mmHg)	2330	3930
CP_MAP_ (mL/min/kg×mmHg)	1550	2660
ECP_Wasserman_ (%mmHg)	8960	10700
ECP_Koch_ (%mmHg)	9000	11600
ECP_Jones_ (%mmHg)	7820	10900
VO_2_/SBP (mL/mmHg)	4.57	7.52

SD: standard deviation; SBP: systolic blood pressure; DBP: diastolic blood pressure; MAP: mean arterial pressure; CP: circulatory power; ECP: exercise cardiac power; VO2: oxygen consumption.

CP is given as a product of VO2 and systolic blood pressure (CP_SBP_) as well as a product of VO2 and mean arterial pressure (CP_MAP_). ECP is presented using the alternative VO2 reference value equations provided by Wasserman et al. [26], Koch et al. [25], and Jones et al. [24] and indexed correspondingly in the table as ECP_Wasserman_, ECP_Koch_, and ECP_Jones_.

Peak VO2, SBP, DBP, MAP, CP, and ECP were lower in female than in male subjects (Table 2). Peak VO2 was dependent on sex, height, weight, PAI, smoking, and fasting insulin levels. Peak SBP was dependent on sex, age, and weight. Peak DBP and MAP were dependent on sex, age, height, and weight. Moreover, DBP was dependent on the use of antihypertensive treatment. In addition, MAP was dependent on fasting insulin levels. The major determinants of peak CP were sex, height, weight, PAI, and smoking. When SBP was used to calculate CP, the waist-to-hip ratio had a small effect on this parameter (Table 3). The estimates of CP that were based on peak VO2 and SBP were very concordant with the estimates based on peak VO2 and MAP (R^2^ 0.96).

As mentioned earlier, we explored several alternative ways to determine ECP (Table 3). All of the derivatives for ECP were dependent on age, height, weight, PAI, and smoking. When ECP was determined using the VO2 reference value equations provided by Jones et al. [24] or Koch et al. [25], or when ECP was expressed as a ratio of peak VO2 and SBP, ECP was also dependent on sex. When ECP was calculated using the VO2 reference value equation provided by Wasserman et al. [26], ECP was independent of sex, but clearly associated with the waist-to-hip ratio. Moreover, fasting insulin levels had a small effect on VO2/systolic blood pressure. Altogether, these factors explained from 21% to 49% of the variation in ECP indices (Table 3).

There was a good concordance between the ECP estimates, when the VO2 reference values used to calculate ECP were generated using the predictive equation provided by Wasserman et al. [26] or Koch et al. [25], the R^2^-value being 0.94. Markedly lower concordance in ECP values was found, when the reference values were generated using the equation provided by Jones et al. [24] or Wassermann et al. [26] (R^2^ 0.67). Moreover, concordance in ECP values was weak when the estimates generated by the predictive equations by Jones et al. [24] and Koch et al. [25] were correlated (R^2^ 0.66).

## Discussion

CPET provides a non-invasive, easily accessible and information-intensive way to obtain parameters for the assessment of cardiovascular status. Low peak VO2 and impaired SBP response in exercise have been shown to have prognostic significance in patients with chronic heart failure (CHF) [27,28].

Peak VO2 alone has been widely used as a substitute for cardiac output (O2 flow). This parameter as an indicator of cardiovascular capacity has the weakness of overlooking cardiac afterload (i.e. systemic peripheral resistance). Peak CP, as a surrogate of cardiac power, has been shown to have better prognostic value in CHF than peak VO2, VO2/HR and peak SBP alone [5,11]. Peak CP correlates closely with cardiac output and cardiac power output at peak exercise [16], making it a relatively good substitute for these parameters provided that the arterial-venous difference in O2 content is invariable [16,27]. The major advantage of CP is that it is a simple and practical index available non-invasively in connection with any CPET without the need for special equipment. CP incorporates information on arterial-venous difference, HR, stroke volume and BP responses at peak exercise [11,12]. Physiologically, CP represents the volume of O2 added to the mixed venous blood by the lungs and transferred to systemic arterial circulation, against a pressure gradient, by the heart [17].

In view of current literature, CP and ECP appear to be promising and useful parameters in the assessment of heart failure risk. To date, the current study is the first in which data on the mean values and normal variation (standard deviations) of CP and ECP is presented in young adults. Therefore, our data, which is based on a cohort of relatively young and healthy subjects of normal aerobic fitness (Table 2) with a low prevalence of cardiovascular medication, is of high value. In order to facilitate the grading of these parameters, we present the lower limits (−2 SD levels below the mean) of peak SBP, DBP, MAP, CP, as well as ECP in Table 4.

There are several illustrative examples in the literature of how CP can be used for the risk assessment of heart failure. In a recent study on 19 women and 156 men aged 53 ± 10 years, CP was decreased to the level of 3198 mL/min/kg*mmHg in patients with CHF, and was the best predictor of outcome when assessed in a multivariable model with peak VO2 [5]. It also appeared that markedly declined CP is probably the best parameter for identifying patients with a particularly poor prognosis, i.e. those with reduced VO2 and reduced BP [5]. The CP- values in the above-mentioned study were well below (−1.5 SD) the mean of the levels observed in the young adults herein. Similarly, in a recent independent study including 631 CHF patients (mean age 58 ± 10 years, 90% males) the mean CP was 2425 mL/min/kg*mmHg, being higher in those who were alive at the end of 3.8 ± 1.4 years follow-up (2500 ± 866) than in those who died (1899 ± 774) [27]. These figures correspond to the −1.8 SD and −2.1 SD limits of the peak mean CP in the current study. Moreover, low peak systolic blood pressure, CP and the presence of exertional oscillatory ventilation have been shown to be superior in their predictive power when compared with traditional CPET-based risk parameters such as peak VO2, O2 pulse and VE/VCO2 slope [27]. A low mean CP of 2605 ± 1198 mL/min/kg*mmHg (−1.8 SD below the mean of the current study) has also observed among 402 CHF patients (aged 55 ± 10 years), and CP was a strong predictor of mortality with higher prognostic value than peak SBP, VO2 and VE/VCO2 slope, irrespective of whether the patients were receiving β-blocker therapy or not [14].

Even better prognostic value for CP was found in the study by Giardiani et al. [11] including 432 patients with adult congenital heart disease. In their study, the patients having a low CP level of below 1476 mL/min/kg*mmHg (calculated here as a product of peak VO2 and MAP) were at a 15.4-fold higher risk of death than those with high CP values. The 1476 L/min/kg*mmHg cut-off level in CP is −2.1 SD lower than the mean CP among the young the adults studied in the present study. Giardiani et al. further showed that CP was the best predictor of mortality during the 4.4-year follow-up. In their study, CP had higher prognostic power than any of the other exercise parameters, being independent of the subjects’ peak VO2 and chronotropic response.

Jaussaud et al. [12] studied 36 patients (aged 60 ± 12 years), who were receiving cardiac resynchronization therapy (CRT). The mean CP of patients before CRT treatment was 1663 ± 494 mL/min/kg*mmHg (i.e. –2.0 SD below the mean CP of the present young adults) and increased to up to 2125 ± 1014 mL/min/kg*mmHg (+ 28%) 6 months after CRT implantation. The authors found that the CP improved more in those patients who responded to therapy (+ 42%) than in those who did not (+18%). Among CRT responders, a rise in CP was associated with a 17% rise in peak VO2, a 5% rise in peak SBP, a 20% rise in maximal WR and a 32% rise in ejection fraction. In addition to a 47% decrease in mean end systolic left ventricular volume, a 26% decrease in mitral regurgitation grade, and a 17% decrease in VE/VCO2 slope was found [12]. As no statistical difference was found in peak VO2, peak SBP, maximal WR, and ejection fraction was found among CRT non-responders, Jaussaud et al. suggested that CP, along with ventilatory response, has the power to differentiate CRT responders from the non-responders [12].

In a previous study of 500 patients with CHF, peak SBP and VO2 - when analyzed as a percentage of predicted peak VO2 – significantly enhanced the risk stratification of heart failure patients [28]. In our opinion, it is preferable to use the percentage of predicted peak VO2 rather than absolute VO2 as a prognostic parameter, because it stratifies the aerobic capacity by age, sex, height, weight, and BMI, i.e. by the factors that are included as variables in the reference value equations.

The idea to use expected values to stratify subject demographics in risk estimation improves the diagnostic accuracy of ECP. In a recent study by Scharf et al. [6] which included 154 CHF patients (88 % of whom were men, mean age of cohort being 51 ± 0.8 years), the predictive power of ECP (expressed as %mmHg) was better than that of aerobic capacity (expressed as % of predicted peak VO2) or peak SBP response alone [6]. ECP was the only independent predictor of mortality when analyzed in a multivariable regression model [6]. ECP <5000 %mmHg (i.e. –2.8 to −3.0 SD below the mean of the present young adults) indicated a 1-year mortality rate of 37% whereas only 2% of the patients having an ECP > 9000 %mmHg (i.e. –2 to −2.2 SD below the level of present young adults) died during the first year of follow-up [6].

In order to provide a reliable basis for characterizing the determinants of CP and ECP, we based the technical performance of our study on international standards and calibrated devices [1,22,23]. We present the numerical data on CP and ECP in several alternative ways, which should help the scientific community to compare the result profiles in their own study settings. Based on the relative normality of our community based cohort, we feel that the current figures of CP and ECP can be considered to represent the average levels of CP and ECP in the general population. The estimates of CP in our study were very concordant, irrespective of whether the BP figures were based on SBP or MAP, rendering both methods of calculation applicable when the studied indices are formed. We recommend the use of the predictive equation provided by either by Wasserman et al. [26] or Koch et al. [25] for the calculation of the ECP estimates, as these equations led to very concordant estimates for ECP. We do not recommend the use of the VO2/SBP ratio to integrate the cardiac output and BP as this parameter showed the highest dependence on subject demographics.

The importance of our preliminary data is emphasized by the fact that the current values are based on almost equal numbers of males and females, while in all of the previous studies described in the above; there is a considerable dominance of male participants. Our study still leaves many unanswered questions, such as whether CP and ECP have prognostic power to predict future cardiac events in unselected cohorts. At this point, we were unable to analyze this aspect, as the current morbidity and mortality rates of our cohort are far too low. However, a prospective analysis of the predictive power of CPET-derived parameters for hypertension, myocardial infarction, stroke and death should be feasible in connection with later cross-sections of the ongoing follow-up on our cohort. We hope that our study encourages other researchers to analyze these parameters further in other patient cohorts, and in healthy, older subjects. The present data is necessary for the compilation of predictive regression equations for CP and ECP in the future.

## Conclusions

As sex, age, subject anthropometrics and lifestyle had an effect on CP and ECP; these factors should be taken into account when the use of CP and ECP is developed further.

## Competing interests

The authors declare that they have no competing interests.

## Authors’ contributions

All authors had full access to the data, and had read and approved the final manuscript. JH developed the concept of this study, involved in acquisition of data, performing the tests, and analysis and interpretation of data, carried out the statistical analysis, and drafted the manuscript. HA was involved in acquisition of data, performing the tests, analysis and interpretation of data, and revising manuscript critically for important intellectual content. KP was involved in acquisition of data, performing the tests, analysis and interpretation of data, and revising manuscript critically for important intellectual content. TL was involved in acquisition of data, handled funding and supervision, and made critical revision of the manuscript for important intellectual content. NHK was involved in acquisition of data, handled funding and supervision, and made critical revision of the manuscript for important intellectual content. JV handled funding and supervision, and made critical revision of the manuscript for important intellectual content. OTR conceived and designed the study, was involved in acquisition of data, handled funding and supervision, and made critical revision of the manuscript for important intellectual content. MK conceived and designed the study, was involved in acquisition of data, handled funding and supervision, and made critical revision of the manuscript for important intellectual content.

## Pre-publication history

The pre-publication history for this paper can be accessed here:

http://www.biomedcentral.com/1471-2261/14/35/prepub
